# The Medical Society of Niagara County

**Published:** 1892-03

**Authors:** 


### THE MEDICAL SOCIETY OF NIAGARA COUNTY.

AT A MEETING of this Society, held December 25, 1891, at the office of Dr. Foote, to take action in relation to the death of Dr. Simeon Tucker Clark, the following-named members were present: Drs. Gould, Ransom, Palmer, Hill, John and E. J. Foote, Hodson, Baker, Johnson, Durand, Ready and Loomis.

Dr. GOULD was called to the chair and spoke as follows :

GENTLEMEN : The sorrowful occasion which has called us together this morning is one that but too sadly and truly emphasizes the words of the Psalmist, "There is but a step between us and death." But a few days since, the robust manly form of our beloved brother was present on our streets and in the homes of many families in our city and vicinity, in the active pursuit of his professional duties, which he loved so well ; in the discharge of which he manifested a zeal and earnestness of purpose that must ever accompany a due sense of the responsibility involved in their faithful discharge. To attainments of a high order he combined the kindlier sympathies of our nature and the pleasing address so essential to win confidence and insure success. His poetic genius, his knowledge and love of music, his extensive and diversified reading were all so many sources from which to draw for the benefit of those entrusted to his care. That these qualifications were appreciated by a discriminating public, the large patronage he enjoyed most fully attests. The intense interest manifested as to the issue, after he was stricken down, by all classes in the community, till death closed the scene and paralysed all hope, is too well known for comment. As a member of our Medical Society, as a professional brother, counselor, and friend, this Society and the profession have met with an irreparable loss.

His cordial greeting and pleasant smile, his friendly aid and valued counsel are ours no more, save in the sacred memories of the past.

Of his literary merits and productions, of his position in the medical college of a neighboring city, where he filled a professor's chair, I leave others to speak, who are better qualified to judge. For the profession it was a sad but pleasing duty to render all the aid in our power to our stricken brother ; and at his bedside, with the beloved wife and children, to mingle our tears in heartfelt sympathy and sorrow ; while in Christian faith we resignedly awaited His will who doeth all things well. And thus, as it were, entering "The valley of the shadow of death," awaiting "the inevitable hour that soon or late must come to all," we accompanied our brother to the cold dark waters of the river which separates time from eternity, and peering with tear-bedimmed vision beyond the confines of time, we caught glimpses of the "Shining shore," and heard the strains of celestial harmonies that came from the angelic escort of the spirit departed to the throne of God and the mansion on high.

Farewell, dear brother ! Be it yours to bask in the sunshine of redeeming love ; to enjoy the rapture of reunion with loved ones gone before. Unfettered by the limitations of time and sense, how shall thy freed spirit glory in the possibilities of an endless life—in the fruition of knowledge that shall come with the never-ending cycles of eternity ; in the joy and friendship of pure spirits that is never broken ; in the ecstasy of delight that comes to the redeemed in the presence of the Christ whose advent to earth was heralded by angelic hosts, proclaiming "Glory to God in the highest, peace on earth, good will to men !" Shall Christmas time on earth be less joyful because of thine absence ? Nay ; let us rejoice to feel that while we are yet subject to the sorrows and disappointments of earth, thou art in His presence where is fulness of joy, at His right hand where are pleasures forever more.

Adieu ! dear brother, not a long, but a brief adieu.
And this our prayer, who still remain,
God be with us, till we meet again.

Drs. Palmer, Ransom, Baker, Johnson, and Loomis also paid feeling eulogies.

The following Committee on Resolutions was appointed: Drs. Kittinger, Palmer, and E. J. Foote, who reported as follows, the report being adopted:

MEMORIAL.

As with noble work well done, our beloved frater and brother co-worker, Simeon Tucker Clark, has folded his willing weary hands in peaceful rest, in the full noontide of ripened manhood, to solve the mysteries of the higher life, glimpses of the bright radiance of which he had so often caught and penned, to brighten the pathway of us who shall come after; as the love-work of a life ever employed to smooth the pillow of the distressed and afflicted has thus suddenly and unexpectedly ended, leaving only cherished memories and saddened hearts;

As a trusted counselor, possessing an educated intellect and ripened experience of priceless value, coupled with a grace, willingness, refinement, tender consideration, and disinterested friendship which few possess and all may emulate, has left his place vacant, and vacant, as well, a place in every heart;

As a bright social link in the burnished chain of fraternity and companionship lies broken and shattered beneath our feet;

As a ready, skilful, helping hand is missed in our necessities;

As a heart, bubbling over with human sympathy, kindness, and good-will to all has ceased to pulsate;

As the voice of a sweet singer of melodious minstrelsy, that sang of purity and love by sweet inspiration from the fountains of life and light, has died away from our listening ears;

As a Christian gentleman, whom we loved, revered, and honored, has left our hearts lonely and desolate;

We are left to sincerely mourn, and to extend our heartfelt sympathies to those dear ones who, by the closer relationship of husband and father, have become so unmeasurably and sorely bereaved by this untimely affliction, and we most sincerely, hopefully, and devoutly commend them to that divine and boundless source of all consolation, comfort, and blessings which he, for whom we are bereaved, so sincerely reverenced and trusted.

But while with them we deeply deplore the irreparable loss they have sustained, we also most reverently desire with them to rejoice for a victory won, a life well done; to rejoice with them in the blessed assurances which are vouchsafed to us all, that —

Not "Down to Emmanus" with blinded eyes,
He walks today
In mortal clay;
But hand-in-hand, in glad surprise,
He walks today
With Christ the way.

*Resolved*, That this memorial be engrossed as a part of the minutes of the Niagara County Medical Society, and that a copy be transcribed and transmitted to the family of the deceased, and that the local papers and the BUFFALO MEDICAL AND SURGICAL JOURNAL be furnished with a copy for publication.

